# Lack of mutations within ST7 gene in tumour-derived cell lines and primary epithelial tumours

**DOI:** 10.1038/sj.bjc.6600418

**Published:** 2002-07-02

**Authors:** V L Brown, C M Proby, D M Barnes, D P Kelsell

**Affiliations:** Centre for Cutaneous Research, Barts and The London, Queen Mary's School of Medicine and Dentistry, 2 Newark Street, Whitechapel, London E1 2AT, UK; Hedley Atkins/Cancer Research UK Breast Pathology Laboratory, Guy's Hospital, Thomas Guy House, St. Thomas Street, London SE1 9RT, UK

**Keywords:** ST7 gene, mutation, tumour suppressor gene

## Abstract

ST7 is a candidate tumour suppressor gene at human chromosome locus 7q31.1. We have performed mutational analysis of ST7 in a wide-range of cell lines and primary epithelial cancers and detected only one missense change in a breast cancer cell line. Other mutations previously found in cell lines and primary tumours were not evident in our analysis. These results imply that another tumour suppressor gene at this locus may be more important than ST7 in carcinogenesis.

*British Journal of Cancer* (2002) **37**, 208–211. doi:10.1038/sj.bjc.6600418 www.bjcancer.com

© 2002 Cancer Research UK

## 

Many strands of evidence point to an important tumour suppressor gene (TSG) on human chromosome 7q31. Frequent deletions within 7q in cytogenetic analyses and a high rate of loss of heterozygosity (LOH) for 7q microsatellite DNA markers are both mechanisms commonly encountered in a wide variety of human tumour types (Zenklusen and Conti, 1996). In addition, functional complementation assays show that microcell fusion of an intact chromosome 7 into cancer cell lines with LOH at 7q31 inhibits tumorigenicity (Ogata *et al*, 1993; Zenklusen *et al*, 1994).

A number of candidate genes for the 7q31 TSG have been described including ST7 (suppression of tumorigenicity 7) formerly known as RAY1 and HELG (Vincent *et al*, 2000). Strong evidence to support ST7 as the key TSG at this locus has recently been reported by Zenklusen *et al* (2001). Using a prostate cancer-derived cell line they have shown that ST7 can suppress *in vivo* tumorigenicity. In addition they described protein-truncating mutations of ST7 in three out of eight cancer-derived cell lines (three out of seven breast cancer and none out of one prostate cancer) and in four out of 10 primary colon carcinomas. The mutations were seen in exon 3 (four cases), exon 5 (two cases) and exon 12 (once) from the 16 exons of ST7.

To explore this further, we have performed mutational analysis of ST7 exons 3, 5 and 12 in a variety of cell lines (cancerous and non-cancerous) and primary epithelial cancer tissues. Mutation analysis was carried out using denaturing high performance liquid chromatography (DHPLC), which is highly specific and sensitive for the detection of genetic mutations, including small deletions, insertions and point mutations (Xiao and Oefner, 2001).

## MATERIALS AND METHODS

Intron-specific primers derived from ST7 cDNA (GenBank AY009152) aligned to genomic sequence were designed to encompass exons 3, 5 and 12 of ST7 (Table 1

**Table 1:**
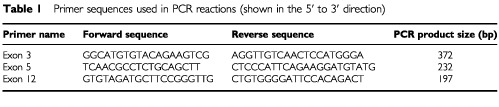
Primer sequences used in PCR reactions (shown in the 5′ to 3′ direction)

). These primers were then used to amplify the DNA from 34 primary breast cancers, 27 sporadic cutaneous squamous cell carcinomas (SCCs), 34 cancer-derived cell lines and 27 non-cancerous cell lines (Table 2

**Table 2:**
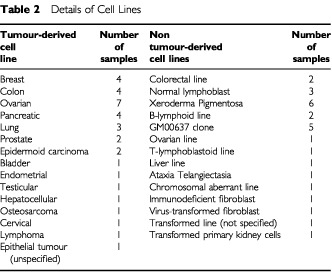
Details of Cell Lines

), by PCR.

Fifty nanograms of DNA were amplified in a 50 μl reaction mixture that contained 200 μM dNTPs, 1×NH_4_ reaction buffer, 1 mM MgCl_2_, 20 pmol primers and 0.5 units Taq polymerase (Bioline, London, UK). Cycling conditions for exons 3 and 5 were as follows: 95°C for 5 min followed by 35 cycles of 95°C for 30 s, 55°C for 30 s and 72°C for 30 s, and a 10 min final extension at 72°C. Cycling conditions for exon 12 were identical except for an annealing temperature of 60°C instead of 55°C.

Prior to DHPLC analysis, heteroduplex formation of the PCR products was carried out by heating for 5 min at 94°C followed by cooling to 40°C at a rate of 0.03°C s^−1^. DHPLC was performed using the Transgenomic WAVE DNA fragment analysis system (Transgenomic, Crewe, UK). The original PCR products of any sample traces for which an aberrant DHPLC elution profile was observed, and all breast and colon cancer samples, were then sequenced directly. In addition, 10% of the samples with normal DHPLC elution profiles were sequenced. The PCR product was purified using a PCR purification column (Qiagen, Crawley, UK), and sequenced using Big-Dye terminator chemistry with the same primers as those used in the original PCR. Sequence analysis was conducted using an ABI 377 automated DNA sequencer and Sequence Navigator software (ABI, Warrington, UK).

## RESULTS AND DISCUSSION

**Figure 1:**
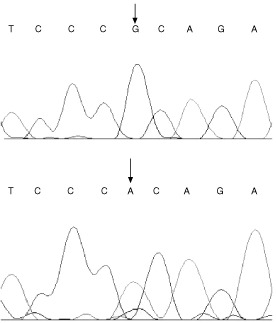
Sequence trace for MDA-MB435 breast cancer cell line (lower trace) showing a G→A substitution at codon 134 of ST7 compared to wild-type (upper trace).

**Figure 2:**
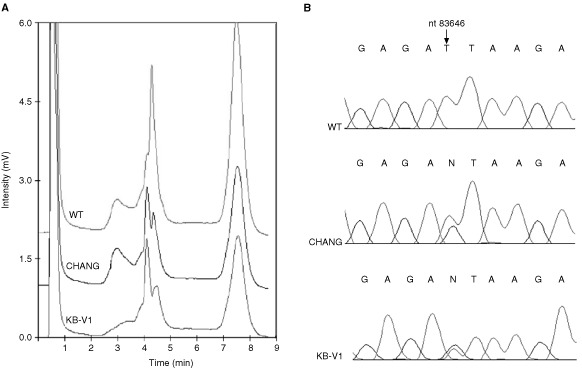
DHPLC and sequencing results of the ST7 intronic polymorphism. (**A**) DHPLC results showing variant traces for CHANG and KB-V1 compared to the wild-type (WT) trace. (**B**) Sequencing showing ST7 intron 3 polymorphism in CHANG and KB-V1 at nucleotide 83646, with reference to human BAC clone CTB-114A6.

**Figure 3:**
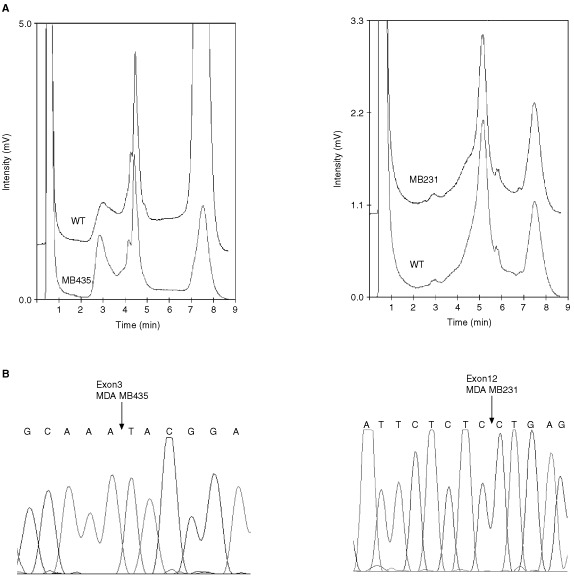
Wild-type profiles of DHPLC and direct sequencing for cell lines MDA-MB435 and MDA-MB231. (**A**) DHPLC traces of ST7 exon 3 for MDA-MB435 (left) and ST7 exon 12 for MDA-MB231 (right). Cell line traces are identical to wild-type (WT) in both cases. (**B**) ST7 sequencing of the cell lines MDA-MB435 (left) and MDA-MB231 (right) for exons 3 and 12 respectively. Arrows show site of mutations described by Zenklusen *et al* (2001) (466-467insT in MDA-MB435 and 1364-1365insT in MDA-MB231), which are not seen in these traces. Nucleotides numbered according to GenBank AY009152 but note 4 bp discrepancy compared to Zenklusen *et al* (2001).

A single missense mutation in the breast cancer cell line MDA-MD435 was detected in exon 5 of ST7 (Figure 1

). This GCA→ACA substitution at codon 134 results in an Ala→Thr amino-acid change. The functional consequence on the ST7 protein of this alteration is not known (Thomas *et al*, 2001). We also obtained variant DHPLC elution profiles for the epidermoid oral carcinoma line KB-V1 and liver cell line CHANG, in the exon 3 PCR products. The profiles from these two samples were similar to each other (Figure 2A

) and subsequent sequencing revealed an identical T → G polymorphism in intron 3 (human BAC clone CTB-114A6; nucleotide 83646) in both cases (Figure 2B). We were unable to detect the single nucleotide insertions that Zenklusen *et al* (2001) reported in the breast cancer cell lines MDA-MB231 and MDA-MB435 by either DHPLC (Figure 3A

) or direct sequencing (Figure 3B). In addition none of the other truncating mutations previously described were found in any of the samples.

Mutational analysis in a wide-range of cell lines and primary epithelial tumours has shown that somatic alteration of ST7 rarely occurs in the exons examined. These results differ markedly from those recently reported (Zenklusen *et al*, 2001). This could be due to the fact that in the previous study all the tumours had been pre-screened for LOH at 7q31, thus increasing the likelihood of detecting a mutation. However, we found a high rate of LOH of 42% at 7q31 in breast tumours from our screening panel (using microsatellite markers D7S522 and D7S677 – data available on request) and would have expected to detect some mutations with this level of LOH. In addition, the cutaneous SCCs examined in this study displayed a lower but still significant rate of LOH at 7q31 of 22%.

Other groups have recently found the same missense alteration in exon 5 in MDA-MB435 that we found (Hughes *et al*, 2001; Thomas *et al*, 2001). They also failed to detect any further coding mutations in all exons of ST7 in a wide-range of neoplastic carcinomas and cell lines, including ovarian, colon, breast, pancreatic and prostate. Our results extend the spectrum of histological types examined for ST7 somatic alterations.

In summary, genetic alteration by nucleotide mutation within ST7 is very rare in epithelial cancers and tumour cell lines. This suggests that if ST7 is the key TSG at 7q31, it is more likely to be inactivated by epigenetic mechanisms or haplo-insufficiency than by direct mutation. Alternatively, one of the other TSG, or an undiscovered gene, at 7q31 is the target involved in carcinogenesis at this locus.
